# A pilot telephone intervention to increase uptake of breast cancer screening in socially deprived areas in Scotland (TELBRECS): study protocol for a randomised controlled trial

**DOI:** 10.1186/1471-2458-14-824

**Published:** 2014-08-09

**Authors:** Julie A Chambers, Ronan E O’Carroll, Alan Cook, Julie Cavanagh, Debbie Archibald, Rosemary Millar

**Affiliations:** Psychology, School of Natural Sciences, Stirling University, Stirling, FK9 4LA UK; Department of Radiology, Ninewells Hospital, Dundee, DD1 9SY UK; Directorate of Public Health, Tayside NHS Board, Kings Cross, Clepington Rd, Dundee, DD3 8EA UK; East of Scotland Breast Screening Service, Ninewells Hospital, Dundee, DD1 9SY UK

**Keywords:** Breast cancer, Screening, Anticipated regret, Telephone reminder, Barriers to breast screening

## Abstract

**Background:**

Breast cancer accounts for almost 30% of all cancers and is the second leading cause of cancer deaths in women in Scotland. Screening is key to early detection. The Scottish Breast Screening Programme is a nationwide, free at point of delivery screening service, to which all women aged between 50 and 70 years are invited to attend every 3 years. Currently over three-quarters of invited women regularly attend screening. However, women from more deprived areas are much less likely to attend: for example in the 3 years from 2010–2012 only 63% of women in the most deprived area attended the East of Scotland Breast Screening programme versus 81% in the least deprived. Research has suggested that reminders (telephone or letter) and brief, personalised interventions addressing barriers to attendance may be helpful in increasing uptake in low-income women.

**Methods/Design:**

We will employ a brief telephone reminder and support intervention, whose purpose is to elicit and address any mistaken beliefs women have about breast screening, with the aim that the perceived benefits of screening come to outweigh any perceived barriers for individuals. We will test whether this intervention, plus a simple anticipated regret manipulation, will lead to an increase in the uptake of breast cancer screening amongst low-income women who have failed to attend a first appointment, in a randomised controlled trial with 600 women. Participants will be randomly allocated to one of four treatment arms i.e. 1) Letter reminder (i.e. Treatment as usual: CONTROL); 2) Telephone reminder (TEL), 3) Telephone reminder plus telephone support (TEL-SUPP) and 4) Telephone reminder plus support plus AR (TEL-SUPP-AR). The primary outcome will be attendance at breast screening within 3 months of the reminder letter.

**Discussion:**

If this simple telephone support intervention (with or without AR intervention) leads to a significant increase in breast screening attendance, this would represent a rare example of a theoretically-driven, relatively simple psychological intervention that could result in earlier detection of breast cancer amongst an under-served group of lower socio-economic women.

**Trial registration:**

Current Controlled trials: ISRCTN06039270. Registered 16^th^ January 2014.

## Background

### The problem

The National Health Service (NHS) Breast Cancer Screening Programme is a national organised screening programme which runs throughout the UK. In Scotland, there are six main breast screening centres including the East of Scotland Breast Screening Service, based in Dundee. Currently, all women aged between 50 and 70 years are called or re-called for screening on a 3-yearly basis; invitations are generated according to General Practitioner (GP) practices. Women aged over 70 years are not recalled automatically but can request screening. Screening is via mammography: a low-dose X-ray used to detect very early tumours, which are too small to be felt. In the East of Scotland region, approximately 95% of women screened have a normal mammogram and 5% are recalled for further tests at an assessment clinic (including specialist X-rays, ultrasound, core biopsies); only 1 in 8 of those recalled will be found to have cancer.

#### Screening uptake

In 2007/8, 16,515 women were invited to attend the East of Scotland Breast Screening service and 12,970 (78.5%) attended, with 611 (5.2%) being referred to the assessment clinic. For the whole region, this attendance rate is higher than the average across Scotland. However, women from more deprived areas are much less likely to attend for screening: for example in the 3 years from 2010–2012 only 63% of women in the most deprived area attended versus 81% in the least deprived. This pattern has changed little since the service began.

#### Benefits of increasing screening

Screening, alongside improvements in treatment, has almost certainly contributed to the fall in deaths from breast cancer in Scotland in the last 20 years (e.g. a 32% reduction between 1990 and 2008 in the 50–69 age group). However, women in the most deprived areas continue to have the worst breast cancer survival rates. Millar [1] predicted that, if screening uptake for all Scottish Index of Multiple Deprivation (SIMD) quintiles could be increased to the highest observed rate, 49–57 extra cancers would be detected in Scotland annually, with an extra 7–33 lives saved. However, it should be noted that this increase could also lead to up to 1316 false positive screens (which turn out to be clear after further diagnostic tests) and 66 over-diagnoses; the latter may result in women receiving treatment for a slow-growing or non-invasive cancer which was unlikely to have caused them any problems if left untreated. Although the numbers of additional cancers detected appear fairly small, the survival rate of patients with early-detected breast cancer via routine screening is approximately double that from cancers detected via other methods [2].

### Existing interventions to increase breast screening uptake

A number of reviews of interventions to increase uptake of breast cancer screening both within and outwith organised screening programmes have been undertaken [3–5]. Few of the interventions covered by these reviews were conducted in the UK, although some did target low-income or socially disadvantaged women. Many of the reviewed interventions are reported to be of poor quality and there is insufficient evidence for the effectiveness of many of the chosen approaches including patient incentives, mass media alone (e.g. public health promotions) and group education. Providing low-income women with detailed medication explanations was not found to be helpful, and may even deter women from attending.

Despite these limitations, there was broad agreement among the reviews that reminders (either letter or telephone) can increase uptake rates amongst all groups. Removing practical barriers, such as making screening more accessible and reimbursing travel expenses may also be helpful to lower socio-economic groups. Having GPs more involved, and providing tailored one-to-one education messages that address individual barriers to screening may also increase uptake in low-income women [3–5]. However, although the latter may be effective, such tailored interventions need direct contact and knowledge of women’s beliefs and attitudes and may be expensive to carry out on a large scale.

### Theoretical background

#### Addressing barriers and concerns

Believing breast cancer screening to be worthwhile, and/or intending to go for screening, does not always translate into actual attendance. The Health Belief Model [6] suggests that the likelihood of engaging in a particular health preventive behaviour, such as attending breast screening, is influenced by the perceived benefits of that behaviour in relation to the perceived barriers to carrying out the behaviour, alongside perceptions of risk and the severity of the targeted illness. Women express a range of barriers to attending breast screening, including lack of knowledge or concerns about the efficacy of screening, anxiety and fear (of the process e.g. pain or the outcome e.g. cancer), embarrassment, practical difficulties such as getting time to attend (e.g. time off work, or from being a carer), cost of transport and difficulties of parking [7]. Many of these barriers are particularly salient to women from socially-deprived areas.

A recent qualitative review of participation in the free, nationwide ‘BreastScreen Australia’ service, which examined both facilitators and barriers to attending screening, found that women expressed both ‘active’ and ‘passive’ barriers to attendance [8]. ‘Active’ barriers tended to be found amongst higher socio-economic women, who had considered the pros and cons of attending and concluded that mammograms were ‘unnecessary’, for instance thinking they might be harmful (e.g. radiation) and that they were not necessarily efficacious (e.g. citing the occurrence of cancers found between screenings). Lower socio-economic women were more likely to express ‘passive’ barriers to non-attendance (e.g. low awareness of the benefits, other demands making it difficult to attend, and practical barriers to attendance). For many women, there wasn’t a single major barrier which prevented them attending, rather there appeared be a cumulative effect of several barriers which tipped the balance towards non-attendance. The authors observed that facilitators to attendance included a belief that the benefits outweighed the barriers to attendance: for example, regular attenders expressed pain as a deterrent as often as non-attenders, but seemed to accept that the benefits of screening outweighed this short-lived negative effect. The report concluded that: ‘the benefits of screening need to be reinforced and women reminded that these outweigh the barriers, even though the downsides may seem more numerous and immediate’ [8], p.62.

In a previous study, in which we aimed to address non-adherence to medication in stroke patients [9], a tailored intervention sought to elicit and address patients’ medication concerns, by providing relevant and tailored information to address any erroneous beliefs they may have held, which could act as barriers to taking their medication. The aim was to ensure that patients’ beliefs in the *necessity* of their medication came to outweigh any perceived *concerns* that they had. We had previously conducted a qualitative study to identify barriers [10], and used the output from this, plus additional input from stroke doctors, to develop a list of potential responses which would help address these concerns and assist patients to overcome any barriers reported^a^. Practical issues of medication-taking, e.g. forgetting, were also addressed by getting patients to state and write down regular plans for taking their medication. This intervention was successful at both increasing the regularity of pill-taking (by 10%), and also reducing concerns about stroke medication, such that at follow-up patient’s views in the necessity of their medication came to outweigh their concerns.

A recent small, pilot study which conducted a brief telephone coaching intervention to address barriers to breast cancer screening uptake in under-served women in the US used a similar approach [11]. This intervention addressed both benefits and barriers regarding screening, for instance, by seeking to increase individuals’ beliefs in the importance of early detection, and provided suggestions on how to overcome personal barriers, such as transportation issues or fear. They found an increase in re-arranged appointments, with almost 95% of women in the intervention group re-scheduling, and 8% more women in the intervention group attended their new appointment compared to the control group (54% versus 46%). The authors suggested that one way of reaching and addressing the psychosocial issues which prevent low-income women from attending breast screening is via brief, tailored interventions conducted within a telephone reminder. Telephone contact requires fewer resources than face-to-face tailored interventions, and could practicably be delivered within an NHS context, particularly if it is targeted only at non-attenders.

We therefore propose that a brief telephone intervention to elicit and address concerns about breast screening, with the aim of ensuring that a woman’s belief in the benefits of breast screening come to outweigh any barriers or concerns, could be effective at increasing screening attendance amongst low socio-economic women.

#### Anticipated regret

Regret is a negative cognitive-based emotion that is experienced when we imagine that the present situation could have been better had we acted differently. It is also possible to *anticipate* regret and thus act to, or prepare to, avoid actually experiencing this unpleasant emotion. Anticipated regret (AR) has been shown to add significantly to the prediction of intentions and prospective health behaviours, over and above the traditional attitudinal components of influential social cognitive theories such as the Theory of Planned Behaviour (TPB) (see review by Abraham & Sheeran [12]). In an AR manipulation in cervical cancer screening, Sandberg and Conner [13] randomised women due to be invited for screening to one of three groups: 1) a control group, 2) a group sent a TPB questionnaire and 3) a group who were asked to complete a TPB questionnaire, as well as answering two AR questions on a Likert-style 7 point scale; “If I did not attend for a cervical smear in the next few weeks I would feel regret”, and “If I did not attend for a cervical smear in the next few weeks, I would later wish I had”. Overall screening attendance was 21%, 26% and 26% in the control group, TPB group and TPB + AR groups respectively (i.e., simply sending out a questionnaire increased attendance by 5%). For those who completed and returned the questionnaire, the attendance rates were 21%, 44% and 65% respectively; thus a subtle AR intervention significantly increased the likelihood of intention to attend screening being translated into actual attendance. This is an impressive effect, given the simplicity, low cost and low intensity of the intervention.

Subtly increasing the prominence of AR in the decision-making process emphasises the aversive emotional consequences of not taking action, and the desire to avoid the negative feeling of regret then motivates people to translate their positive intentions into action, because failing to act may be associated with unpleasant emotions. There is also evidence that using interventions which target emotions rather than cognitive processes (such as education or providing detailed information) may be more effective with low-income groups [14]. It is likely that higher socio-economic individuals may be better able to evaluate facts or evidence, whereas those from lower socio-economic groups may be more prone to use emotions to guide their decision-making. As illustrated by Sandberg and Conner [13], the concept of AR seems particularly relevant to the context of cancer screening, as not taking part in screening and then later being diagnosed with cancer is likely to lead to marked feelings of regret.

Based on the approach of Sandberg and Conner [13], we are currently testing an AR intervention in a large-scale, questionnaire-based study with 60,000 people invited to take part in the Scottish Bowel Cancer Screening programme where patients are sent Faecal Occult Blood Testing kits, to complete at home [15]. We are predicting that AR will be related to a greater uptake in test kit return across all socio-economic groups.

We therefore propose that a similar AR manipulation may also increase breast cancer screening rates among low socio-economic women.

### Pilot studies

#### Factors associated with poor uptake in low-income women in Tayside

A small qualitative survey, aimed at eliciting factors associated with low uptake in low-income women, was previously conducted in NHS Tayside [16]. Women from two GP practices in low-income areas who had failed to attend their most recent screening appointment were invited to attend focus groups in a local community centre; participants were reimbursed £10 for expenses/inconvenience. Eleven women agreed to take part in 2 focus groups and 7 attended. Some of the women suggested they had been screened on at least one previous occasion; thus this small, self-selected sample is unlikely to represent those most resistant to attending breast screening. Millar therefore proposed that “future work to encourage non-attenders to share their reasons for not attending utilising other methods such as telephone interview or questionnaire may prove effective in gaining the views of a wider group”.

Nonetheless barriers to breast screening emerging from the focus groups mirrored those found in other research e.g. [7]. These included: fear and anxiety (e.g. of the process, potential outcome, radiation levels, waiting for results), life issues (e.g. being carers or working and finding it hard to get away, poor health) and access (e.g. transport, parking, location, weather). Participants also suggested that reminders, peer-led support and advice, and more mobile units might be helpful. The themes from the focus groups were reviewed with staff at the breast screening service, and there was frequent concordance with the reasons expressed by the non-attending women. The barriers generated from this research will be used as the basis for eliciting and addressing barriers and concerns in the current study.

#### Anticipated regret (AR)

As outlined above, we are currently conducting an AR intervention study in colorectal cancer screening [15]. This research will test whether adding two AR questions to a simple questionnaire significantly increases screening uptake in people from all social deprivation categories. In a different context, we have also shown that the simple AR manipulation that we propose using here led to a significant increase in intention to become an organ donor [17] and self-reported organ donor registration [18].

### Aims

Our aim is to elicit and address barriers and facilitators to breast screening attendance in low socio-economic women via a brief, personalised, telephone intervention, aimed at increasing screening uptake. We will also test whether asking questions about anticipated regret (AR) leads to additional increases in screening uptake; and examine whether this brief intervention is feasible and acceptable to low-income women.

### Research questions

1. Is a simple, telephone reminder intervention, aimed at eliciting and addressing the barriers and concerns of women in socially-deprived areas regarding attending screening at the East of Scotland Breast Screening Service, feasible and acceptable to participants?
2. Can this brief, telephone intervention increase uptake in non-attenders from socially-deprived areas?
3. Does adding anticipated regret to the telephone intervention have any additional benefit in terms of increasing uptake of screening in non-attenders?

## Methods/Design

**Figure 1:**
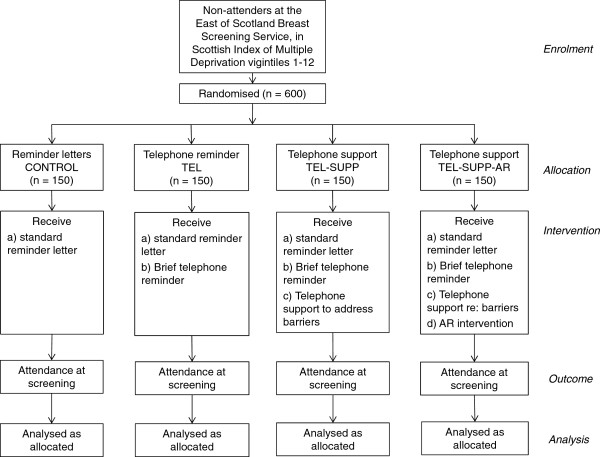
**CONSORT diagram of study design.** Note: Scottish Index of Multiple Deprivation vigintiles 1-12 represent the highest 60% of areas of deprivation in Scotland (based on postcode area).

We will adopt a simple, between-groups, four-arm prospective RCT design. The intervention will be targeted at those who are due to be sent a reminder letter i.e. non-attenders at a scheduled appointment. The four arms are: 1) Letter reminder (i.e. Treatment as usual: CONTROL); 2) Telephone reminder (TEL), 3) Telephone reminder plus telephone support (TEL-SUPP) and 4) Telephone reminder plus telephone support plus AR (TEL-SUPP-AR). The CONSORT diagram is shown in Figure 1.

### Setting

A single-centre trial based at the East of Scotland Breast Screening Centre, located within NHS Tayside in Dundee, Scotland.

### Ethical approval

Ethical approval has been granted by Tayside NHS Board, East of Scotland Research Ethics Committee REC 1 (ref. no. 13/ES/0128).

### Recruitment

Participants will be selected from those receiving an appointment for breast screening who do not telephone to cancel or re-arrange their appointment and who fail to attend their appointment before the reminder letter is due to be sent. Sampling will be from the lists of women for whom reminder letters are generated. We plan to target women from lower socio-economic areas, specifically women from Scottish Index of Multiple Deprivation (SIMD) vigintiles 1–12 (representing the lowest 60% of socio-economic areas in Scotland)^b^. The Scottish Index of Multiple Deprivation assesses deprivation levels for small geographical areas, based on income, employment, health, education, skills and training, housing, geographic access and crime. Based on the numbers of women who did not attend their designated appointment at the East of Scotland Breast Screening Service during 2012, we estimate that in 6 months we could recruit approximately 1,030 women. Currently the screening database does not include a telephone number for all women. We will therefore seek to obtain this information from other data collections (see Telephone numbers below). We would achieve our target for randomisation of 600 women, who are contactable by telephone, providing we are able to obtain approximately 58% of telephone numbers. We will seek to obtain telephone numbers before randomisation, so there is no bias in the Control group (for whom we do not require telephone numbers).

#### Telephone numbers

Telephone numbers are not currently held on the screening database. Some patients may have telephone details on the local hospital administration system and this will be searched in the first instance for phone numbers. Subsequently online and past and present paper telephone directories will be used to try and obtain numbers, where possible. We would therefore aim to include significantly more participants than required for analysis to account for the likelihood of having a large number of non-contactable women, and have chosen the SIMD vigintiles used to reflect this need. This would be monitored throughout the project, and if necessary (for instance if we were able to obtain more or fewer phone numbers than predicted), the SIMD range could be contracted or widened, as appropriate. The feasibility of obtaining telephone numbers is an important secondary outcome of this proposed research.

### Inclusion criteria

Sampling will be via the list of women who are due to be sent a reminder letter for a missed breast screening appointment, and who have not telephoned to cancel or rearrange their appointment. From this list, we will include all women residing in SIMD vigintiles 1–12, for whom telephone numbers can be obtained.

### Exclusion criteria

We will exclude participants who appear to have difficulty understanding the verbal information presented about the study and/or appear to be incapable of deciding to give consent, when telephoned. (If there is any doubt about this, then the participant will be excluded.) This will include people who have difficulty understanding the English language. Women expressing a desire not to take part due to existing illness which may preclude their attending breast screening (e.g. breast cancer) would also be excluded. There are no other exclusion criteria.

### Informed consent

To preserve anonymity, no information will be given out about the nature of the phone call, until the Research Fellow is certain they are talking to the correct person. Verbal informed consent will be obtained at the beginning of the telephone support intervention, for those participants who affirm they are willing to be asked a few questions about breast screening. (Those who decline to answer any questions will be deemed to have refused consent and will not be contacted further.)

First, the purposes of the study will be read from a Patient Information Sheet. It will be made clear that the researcher is completely independent of the breast screening service and the participant's decision to participate (or not) will not affect their future treatment and that confidentiality of all their answers will be ensured. The participant will then be asked whether they consent to take part in the intervention.

Although we are not seeking written consent from intervention participants, patients' responses to the consent question will be recorded on a datasheet, and those refusing consent will be excluded from the study and will have no further contact from the researchers. Consent to tape-record the interview will also be sought; however, refusal to be recorded will not result in exclusion. Prospective participants who feel unable to decide immediately whether to take part will be given the option to be called back later, once they have had time to consider. They will also be referred to an independent researcher, if they would prefer to talk to someone else regarding the research. Participants who feel unable to decide will also be given the option of being posted the Information Sheet; and a follow-up telephone call will be arranged, to see whether they have decided to take part.

Due to the design of our study, in which it is imperative that the telephone intervention is conducted as close to the posting of the reminder letters as possible to ensure parity of timing with the control and three telephone intervention groups, and to reduce the possibility that participants will already have rearranged their appointment before being telephoned, we are unable to seek written consent from participants. However, we believe that we have addressed any concerns about this by having the Research Fellow read the Information Sheet to all participants, as well as allowing prospective participants the opportunity to take time to reflect on taking part, speak to an independent person and/or to be posted the information sheet.

We are not seeking consent to collect follow-up attendance data from any participants for the following reasons: this would be impracticable for the control group; and for the three telephone groups, informing them that we would be checking their future attendance would in itself constitute an intervention, thereby confounding our true intervention, and precluding direct comparison with the control group. This would have the effect of rendering our results scientifically meaningless. Not seeking consent for collection of data on screening uptake is an approach previously used in a number of previous studies with cancer screening, all of which have gained ethical approval from UK National Health Service (NHS) Ethics Research Committees (i.e. colorectal cancer screening [15, 19]; cervical cancer screening: [13]).

We feel this approach is justified in the current research for the following reasons: (a) no harm will come to the participants from our collecting screening data, (b) our research cannot be practically carried out if we had to receive written informed consent from all participants, (c) the potential benefits to the NHS (i.e. determining effective methods of increasing breast screening in Scotland) outweigh any cost, (d) the East of Scotland Breast Screening Service already have access to the attendance data and (e) no individual identifiable results will be recorded in the database for analysis.

All data for analysis will be completely anonymised and confidential. Staff at the East of Scotland Breast Screening Centre will not see any data from the telephone intervention, and staff at the University of Stirling will not record any personally identifiable information on patients alongside the intervention data: the two datasets will be linked by an anonymised unique identifying code. We therefore believe we have taken all necessary steps to eliminate any risk to participants in the current study which may arise from their not giving informed consent for collection of attendance data. Informed consent will be obtained for participation in the telephone support intervention.

We have obtained full UK NHS IRAS ethical approval for this approach (Tayside NHS Board, East of Scotland Research Ethics Committee REC 1 (ref. no. 13/ES/0128).

### Design

We will adopt a simple, between-groups, four-arm prospective RCT design. Participants will be sampled from women who have failed to attend their recent appointment for breast screening. Women who have telephoned to decline or rearrange their screening appointment will be excluded. The intervention will be targeted at lower income women i.e. those from SIMD vigintiles 1–12.

The four treatment groups are: 1) Letter reminder (i.e. Treatment as usual: CONTROL); 2) Telephone reminder (TEL), 3) Telephone reminder plus telephone support (TEL-SUPP) and 4) Telephone reminder plus support plus AR (TEL-SUPP-AR).

#### Letter reminder group (CONTROL)

Participants randomised to the Control group will receive the reminder letter only, as current practice. This will be sent from the East of Scotland Breast Screening Centre.

#### All telephone groups (TEL, TEL-SUPP, TEL-SUPP-AR)

All telephone groups will receive the standard reminder letter in the same manner as the Control group. Participants in each of the three telephone groups will also be telephoned by the Research Fellow from the University of Stirling within two weeks of the reminder letter being posted. Telephone calls will be tape-recorded in order to check the fidelity of the intervention, and also to ensure accuracy of the participant’s answers recorded on the check-list. Participants who opt not to allow their calls to be recorded will still be able to take part in the intervention. Participants who do not answer or are not at home when telephoned, would be called on a maximum of 5 occasions, after which they would be recorded as non-contactable.

#### Telephone reminder group (TEL)

The telephone reminder (TEL) will be a simple telephone call to remind non-attenders that they did not attend their scheduled appointment and provide information on how they can rearrange this appointment. Women wishing to rearrange their appointment will be given the option of being transferred directly to the appointments service at the Breast Screening Centre in Dundee.

#### Telephone support groups (TEL-SUPP and TEL-SUPP-AR)

Participants who are allocated to the telephone support intervention arms (TEL-SUPP and TEL-SUPP-AR) will be told that we are trying to understand why some women do not take up their invitation to attend for breast screening when invited and asked whether they would be prepared to answer some questions about breast screening. Those declining at this point would be assumed to have not given consent and would not be contacted further. The Patient Information Sheet will then be read out to prospective participants. This explains the nature and purpose of this research and reassures participants about the confidentiality and anonymity of their responses. They will be asked if they have any questions about our research and, if yes, will also be given the option of being able to talk to a Health Psychologist, who is independent of this research. They will be asked whether they agree to take part now (Yes/No), or whether they would prefer to be phoned back later.

In both telephone support arms (TEL-SUPP and TEL-SUPP-AR), patients will be asked to describe any reasons they had for not taking up the invitation to attend their appointment, and where appropriate, any barriers they mention will be addressed using a pre-specified list of responses, which will be generated from previous research (e.g. other women have mentioned that difficulty, but they managed to get over it by…. (How) could that work for you?’). Any patient queries or concerns about the process of breast screening will also be addressed, using responses from existing materials. Whether or not specific barriers are mentioned by individuals will be recorded on a check-list of barriers generated from the existing research and previous telephone interviews. Any additional barriers mentioned will be added to this check-list.

Participants in the TEL-SUPP and TEL-SUPP-AR groups would also be asked to say whether they now intended to make an appointment to attend for breast screening: ‘Do you intend to make an appointment for breast screening?’ ‘Yes’, ‘No’, ‘Maybe’ or ‘Don’t know’.

Participants will be reassured throughout that it is their choice whether or not to take up their invitation to attend breast screening, but that the researcher is there to seek their views on attending and to provide them with additional help and information, should they wish to make another appointment to attend.

In order to check the acceptability and feasibility of the telephone support intervention, participants will be asked whether they minded being phoned up about breast screening, and also whether they found the telephone call helpful in addressing any concerns, queries or issues they might have regarding attending breast screening (‘Yes’, ‘No’, ‘Maybe’ or ‘Don’t know’).

It is envisaged each phone call would last 5–10 minutes, although some may take longer, if a participant has a lot of issues she wants to discuss, and some would be shorter, if the participant does not mention any barriers or does not wish to take part. Women who express a desire to rearrange their appointment will be given the option of being transferred directly to the appointments service at the Breast Screening Centre in Dundee at the end of the telephone intervention.

#### Telephone support plus anticipated regret only (TEL-SUPP-AR)

In the TEL-SUPP-AR group, participants will receive exactly the same intervention as the TEL-SUPP condition, with the addition of two questions relating to Anticipated Regret, ‘If you didn’t make another appointment to attend for breast screening, would you later wish you had?’; ‘If you didn’t attend for breast screening, would you later regret it?’ Participants would be asked to respond ‘Yes’, ‘No’, ‘Maybe’ or ‘Don’t know’. These questions would be asked at the end of the support intervention, immediately before the question relating to intention to attend screening.

### Training materials

Detailed training materials will be produced by the Research Fellow, based on the techniques and materials used in the telephone intervention. This will enable the intervention to be subsequently conducted by NHS staff.

### Sampling and randomisation

Participants will be selected from those receiving an appointment for breast screening who do not telephone to cancel their appointment and who fail to attend or to re-arrange their appointment before the reminder letter is due to be sent. Sampling will be from the lists of women for whom reminder letters are generated. Postcodes will be used to derive Scottish Index of Multiple Deprivation (SIMD) vigintiles, and women who are from a SIMD vigintile between 1 and 12 inclusive (where 1 represents the most deprived areas), for whom telephone numbers can be obtained, will be randomised to treatment arm in a 1:1:1:1 ratio.

In order to achieve a balance between treatment groups, randomisation will be carried out by a member of the research team who is independent of the intervention on a record by record basis using minimisation via the MINIM software program [20] with age band (50–56, 57–63, 64 or older) and SIMD quintiles (i.e. 1, 2, 3 equivalent to vigintiles 1–4, 5–8 and 9–12) as the minimisation variables. The minimisation algorithm allocates the first record randomly and subsequent participants are allocated to minimise the imbalance between groups on the minimisation variables. It is currently the best available method of achieving randomisation whilst “ensuring excellent balance between groups for several prognostic factors, even in small samples” [21]. To ensure that the researcher does not influence the randomisation process, participants will be entered into the MINIM software sequentially, according to their order in the reminder letter list and the researcher will also remain blind to the identity of each treatment group (i.e. by randomising only to A, B, C, or D) during the randomisation process. The same researcher will collect the three-month follow-up data and carry out the data analysis and will continue to remain blind to the identity of each treatment group until this is complete.

### Data collection and security

The Research Fellow will require access to patients' personal details to enable contact by telephone. Only the minimum information required for contact will be recorded. Any personal information will be stored separately from data collected at interview. All information will be kept either on a secure network computer and/or in a locked filing cabinet, in a locked office, at the University of Stirling.

A unique anonymised identifier will be used to enable matching with the follow-up attendance data. The follow-up attendance data will be collected from the screening database on-site at the Breast Screening Centre, 3 months after the reminder intervention. The anonymised data from the telephone interventions and attendance screening data will be stored on a secure password-protected network server computer and analysed by the Research Fellow at the University of Stirling. Data integrity will be enforced by cross-checking with the recorded interview after the intervention is complete and by valid value and range checks at the time of data entry. Data storage systems at the University are built to be resilient to failure; their locations have secure physical entry and protected power supplies and are guarded by fire and intruder alarms. Data will be kept for a period of 10 years, in line with University Policy, after which it will be securely destroyed.

### Primary and secondary outcomes

The primary outcomes, collected at 3-month follow-up, will be whether or not each patient makes another appointment for breast screening and whether or not they attended screening within 3 months of receiving their reminder (letter or telephone call). Previous attendances and previous failures to attend (attendance history) will also be collected at this time. Secondary measures for the two telephone support arms, collected during the telephone interview, are intention to make another appointment, anticipated regret, number of barriers to breast screening mentioned by participants, and specific barriers reported. In addition the percentage of participants for whom telephone numbers are obtainable at the outset is an important secondary outcome.

### Analysis

Our primary analysis will be chi-square to detect differences in the proportion of respondents who attended breast screening within 3 months of the reminder letter or call as a function of the 4 arms (control, TEL, TEL-SUPP or TEL-SUPP-AR). This will be followed by logistic regression of attenders versus non-attenders in the four groups, controlling for potential between-arm differences in age, social deprivation (SIMD) and history of previous breast screening attendance. Our primary analysis will be on an ‘Analysed as Allocated’ basis, i.e. including all participants, whether or not they were successfully contacted by telephone and/or agreed to take part in the study. Secondary analysis will be conducted for those agreeing to and participating in the intervention (TEL-SUPP and TEL-SUPP-AR groups only) to test the mediating and moderating roles of reported barriers, intention and AR in uptake of screening after reminder.

### Power calculations

A power calculation indicates that a sample of 600 participants would provide 80% power (at the 5% level) of detecting an increase of 11% in the TEL-SUPP and TEL-SUPP-AR groups versus no increase in the Control group and a 3% increase in the telephone reminder group (TEL), assuming a base attendance rate of around 69% (as currently present in vigintiles 1–12). This would also equate to 67% power (at 5% level) of detecting an increase in uptake of 9% spread across the 3 telephone intervention arms versus the Control group.

### Evaluation

The effects of the intervention will be evaluated via measurement of the primary and secondary outcome variables listed above.

#### Process evaluation

As this study is based on a one-off telephone call no process evaluation is required.

### Timetable

This is a 17-month project. Months 1–3 will be spent designing the telephone support intervention and developing the barriers checklist and recruitment procedures. During this period we shall obtain IT approval for the Research Fellow to access the required databases, as well as Caldicott approval for the data extraction. The Research Fellow will also gain an NHS Tayside ‘letter of access’, which permits access to the required data systems, for the duration of the study.

Months 4–9 will be spent conducting the telephone intervention. We plan a sample size for analysis of 600; with 450 women randomised to the three telephone interventions, this equates to around 75 telephone calls per month.

We require a three-month lag after the intervention to collect data on screening attendance. Therefore, months 10–15 will be spent collecting the data on screening attendance and analysing the data. The training materials will also be developed during this time. In months 16–17, we will produce a final report and a paper of the main results for publication, and hold presentations on the results for NHS Tayside staff.

## Discussion

It is estimated that increasing breast cancer screening uptake to the level found in higher socio-economic women (i.e. approximately 80%) across all socio-economic groups could translate into approximately 49–57 additional cancers diagnosed across Scotland annually, with an extra 7–33 lives saved. This equates to a 20% increase in the lowest uptake groups in Dundee. If this simple telephone support intervention (with or without AR intervention) leads to a significant increase in breast screening uptake, this would represent a rare example of a theoretically-driven, relatively simple psychological intervention that could result in earlier detection of breast cancer amongst an under-served group of lower socio-economic women.

This project has the full support of both the Scottish Breast Screening Programme Board and the Detect Cancer Early Programme. If the current trial proves a clear advantage of the AR condition, it has the potential for implementation in the future National screening programme, e.g. by adding the AR questions to the reminder letter. If the results of the trial are positive, they will be communicated immediately to Scottish Breast Screening Programme Board, who will then advise the Scottish Government Department of Health on implementation.

## Endnotes

^a^For example, patients were sometimes concerned about potential long-term dependence or negative effects of taking their medication; reassuring them that the medication they were currently taking was not addictive, had been used extensively over many years, and had not been found to have serious side-effects over that time could help alleviate concerns and make patients less reluctant to take their medicine.

^b^At a national level for Scotland it is estimated that this split would represent current screening attendance rates of approximately 69% (vigintiles 1–12) versus 80% (vigintiles 13–20).

### Trial sponsor

The trial sponsor is Ms Julia Campbell, University of Stirling, Stirling, UK, FK9 4LA.
